# Mach bands explained by response normalization

**DOI:** 10.3389/fnhum.2014.00843

**Published:** 2014-11-04

**Authors:** Frederick A. A. Kingdom

**Affiliations:** McGill Vision Research, Department of Ophthalmology, McGill UniversityMontreal, Quebec, Canada

**Keywords:** Mach bands, brightness illusions, response normalization, feature coding, multi-scale filtering

## Abstract

Mach bands are the illusory dark and bright bars seen at the foot and knee of a luminance trapezoid. First demonstrated by Ernst Mach in the latter part of the 19th century, Mach bands are a test bed not only for models of brightness illusions but of spatial vision in general. Up until 50 years ago the dominant explanation of Mach Bands was that they were caused by lateral inhibition among retinal neurons. More recently, the dominant idea has been that Mach bands are a consequence of a visual process that generates a sparse, binary description of the image in terms of “edges” and “bars”. Another recent explanation is that Mach bands result from learned expectations about the pattern of light typically found on sharply curved surfaces. In keeping with recent multi-scale filtering accounts of brightness illusions as well as current physiology, I show however that Mach bands are most simply explained by response normalization, whereby the gains of early visual channels are adjusted on a local basis to make their responses more equal. I show that a simple one-dimensional model of response normalization explains the range of conditions under which Mach bands occur, and as importantly, the conditions under which they do not occur.

## Introduction

**Figure 1:**
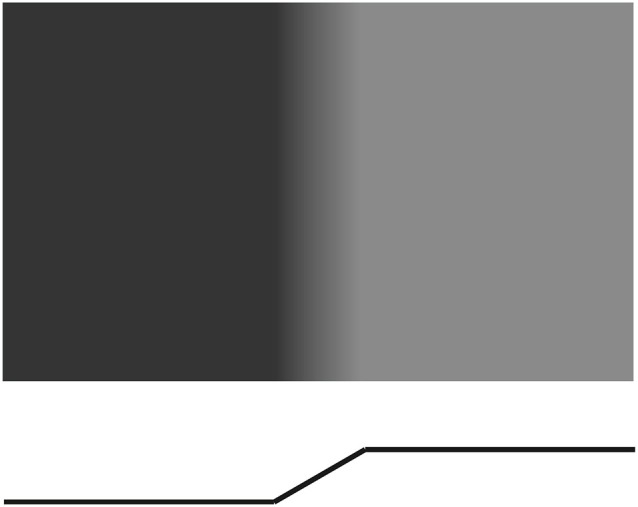
**Top: A trapezoidal edge showing Mach bands**. Bottom: schematic of luminance profile.

**Figure 2:**
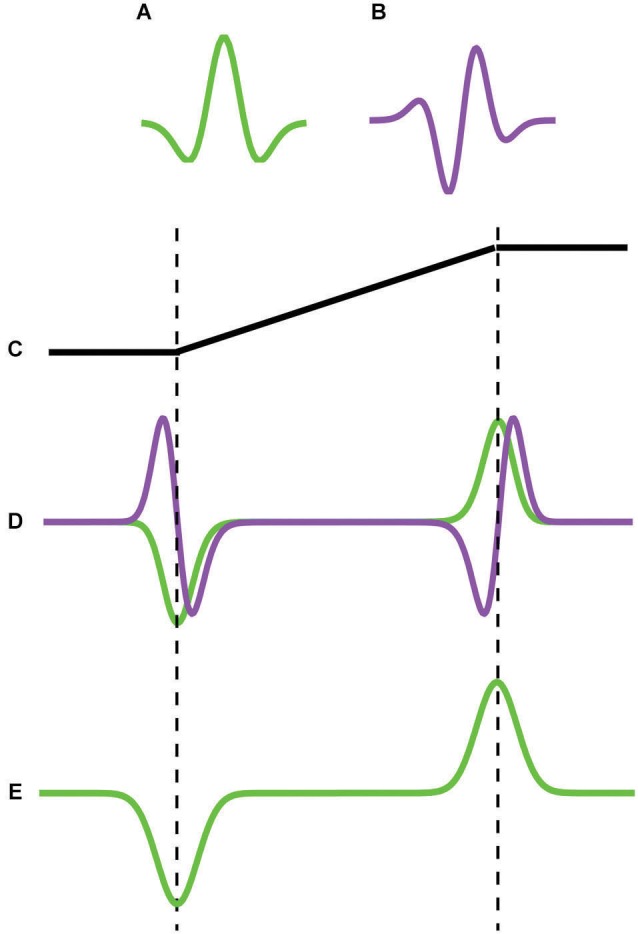
**Schematic of the principle behind Feature models of Mach bands that employ even- and odd-symmetric filters**. **(A)** even-symmetric **(B)** odd-symmetric filter. **(C)** trapezoidal edge profile. **(D)** response of even (green) and odd (purple) filters. The even-symmetric filter gives a strong response at the foot and knee of the trapezoid whereas the odd –symmetric filter gives zero response. **(E)** bars are signaled at the trapezoid’s foot and knee.

**Figure 3:**
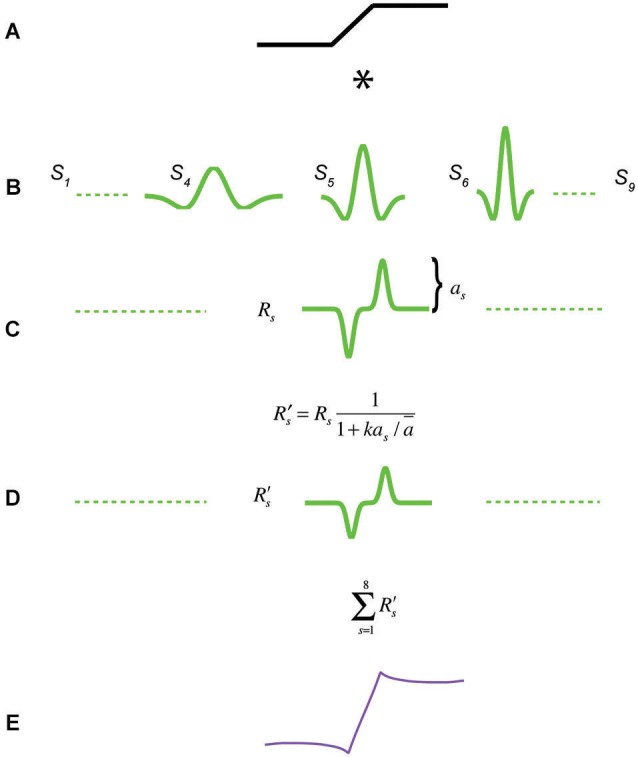
**Response normalization model of Mach bands**. A trapezoidal edge in **(A)** is convolved with a bank of 9 even-symmetric filters at octave scale intervals in **(B)**, to produce the responses in **(C)**. Each response is subject to a normalization function that renders the response amplitudes across scale more equal, resulting in **(D)**. All filter responses are then summed to produce the model response in **(E)**.

Ernst Mach was the first to report the illusory dark and bright bars on a luminance trapezoid that now bear his name (Mach, 1865; translated by Ratliff, 1965)—see Figure 1. As with other illusory brightness phenomena, numerous explanations for this intriguing phenomenon have since been proposed (the earlier explanations are reviewed by Ratliff (1965) and more recent ones by Pessoa (1996) and, briefly, by Kingdom (2011)). Mach proposed that the bands were seen at the peaks and troughs in the second derivative of the luminance profile, and conjectured that this was due to reciprocal interactions between neighboring retinal cells, in other words “lateral inhibition”. As noted by Wallis and Georgeson (2012), a similar conclusion was reached by investigators who began the re-examination of the phenomenon some 60 years ago (Burnham and Jackson, 1955; O’Brien, 1958; Charman and Watrasiewicz, 1964; Thomas, 1965). Indeed, if one convolves a trapezoidal function with an even-symmetric bandpass filter, such as a model of a retinal ganglion or lateral-geniculate-nucleus (LGN) cell, one observes a dip at the foot and a bump at the knee of the trapezoid. However, any *single* bandpass filter account of Mach bands is problematic for two reasons. First, if the filter is dc-balanced, it incorrectly predicts the same brightness either side of the ramp (Figures 2, 3). Unbalancing the filter gets round this problem but a second problem remains: the single-filter response shows the largest Mach bands at step edges, where none occur (Fiorentini, 1972; Tolhurst, 1972; Ross et al., 1981; Ratliff, 1984). That Mach bands are not seen at a step edge is arguably a defining constraint of any model of the phenomenon. However the observation itself is not always appreciated, so given that the appearance of a step edge is a matter of some importance it will be considered in detail here.

The absence of Mach bands at step-edges prompted Tolhurst (1972) to suggest that Mach bands result from inhibitory interactions between “edge” and “bar” detectors. At a step edge, Tolhurst argued, strongly stimulated edge detectors inhibit weakly stimulated bar detectors, preventing any illusory Mach bands from appearing. On the other hand with a trapezoid, the optimally excited edge detectors are found in the center of the ramp while the optimally excited bar detectors are found at the foot and knee of the ramp. Because the two are spatially separated, Tolhurst argued, there is less inhibition of the bar detectors by the edge detectors, with the result that the illusory bands appear. Tolhurst’s account of Mach bands in terms of interactions between edge and bar detectors anticipated the spate of models termed here “Feature” models that emerged in the 1980s, all of which attempted to provide an account of Mach bands. The models include MIRAGE (Watt and Morgan, 1985; Morgan and Watt, 1997), the local energy model (Morrone and Burr, 1988; Ross et al., 1989), MIDAAS (Kingdom and Moulden, 1992) and most recently the N2+1+ model (Wallis and Georgeson, 2012). Inspired by Marr’s (1982) notion of the primal sketch, Feature models are based on the idea that early vision generates a sparse, binary, symbolic description of the image in terms of “edges” and “bars”. The symbolic edge-vs.-bar description is generated using rules that interrogate the responses of linear bandpass filters tuned to different scales and/or orientations. In some Feature models (e.g., MIRAGE, MIDAAS) only even-symmetric filters are employed, and the edge-vs.-bar description is based on the shape of the filter response profiles: “edge” if the profile is odd-symmetric, “bar” if even-symmetric. In other models (e.g., local energy, N2+1+) both even- and odd-symmetric filters are employed, and the edge-vs.-bar description is based on the relative activity of the two types of filter: “edge” if the odd-symmetric response is dominant, “bar” if the even-symmetric response is dominant. Although the details of the filters employed, the manner in which they are combined (if at all) and the rules for interpreting their outputs differ between Feature models, all share the key idea that the foot and knee of a trapezoid elicits a relatively strong response in an even-symmetric bandpass filter, and this is interpreted as indicating the presence of a bar. Figure 2 is a schematic of the *principle* behind those Feature models that employ odd- and even-symmetric filters, but it must be understood that important details of the model implementations are left out of the figure. In the figure it is the relative strength of the even- compared to odd-symmetric filter responses at the foot and knee of the trapezoid that is interpreted as “bar not edge”. On the other hand, the opposite obtains for a step edge, where the responses of the odd-symmetric filter are dominant, leading to the “edge not bar” interpretation. One advantage therefore of Feature models over lateral inhibition is that Feature models provide an explanation of not only why Mach bands are seen in trapezoids, but crucially why they are not seen at step edges.

In this communication the case will be put that in spite of the superiority of Feature models over earlier lateral inhibition models, there is a simpler and more parsimonious explanation of Mach bands: response normalization. Response normalization is the canonical physiological process whereby the responses of neurons are normalized with respect to the local average activity of other neurons in the vicinity (reviewed by Carandini and Heeger, 2012). Response normalization is closely related to the concept of contrast normalization, where physical contrast rather than neural response is used to model the normalization process (Carandini and Heeger, 2012). One of the effects of response normalization is to make more equal the filter response magnitudes of neurons in different parts of the image, and it is this response equalization property that is argued here to be the cause of Mach bands.

The inspiration for considering response normalization as a possible cause for Mach bands comes from a recent class of model aimed at providing low-level accounts of a variety of brightness illusions. This class of model combines multi-scale filtering with response (often termed contrast) normalization (Blakeslee and McCourt, 1999; Dakin and Bex, 2003; Blakeslee et al., 2005; Robinson et al., 2007; Otazu et al., 2008). However only one of these models, the contextual interaction model of Otazu et al. (2008), has been applied to Mach bands. While the model successfully predicts Mach bands in a trapezoidal edge, it also predicts Mach bands at a step edge (Otazu, personal communication), and it remains to be determined whether it can account for the observed differences in the magnitude and width of Mach bands across the variety of stimuli that are considered here.

This communication comprises a modeling exercise that demonstrates how a simplified model of response normalization that instantiates directly its response equalization property provides an account of not only the circumstances in which Mach bands occur, but just as importantly when they do not occur. The model is one-dimensional (1D) and is applied to the 1D luminance profiles of various trapezoidal and related stimuli. A brief report of the model has been given elsewhere (Kingdom, 2013).

## The model

Figure 3 outlines the model. Each 1D stimulus is convolved with a bank of 1D second-derivative-of-Gaussian (even-symmetric) filters, whose gains are such as to give the same peak-amplitude response to a step-edge, in keeping with the properties of retinal ganglion cells (Croner and Kaplan, 1995) and early-stage filtering models based on efficient coding (Graham et al., 2006). The equation for the filters is:

(1) $$F\left(x;\sigma \right)=-\frac{1}{\sigma }\left(\frac{x^{2}}{\sigma^{2}}-1\right)\exp\left(\frac{-x^{2}}{2\sigma^{2}}\right)$$

where *x* is position and σ the standard deviation of the underlying Gaussian. The model employs 9 filters of different scale (only three are shown in the figure), achieved by setting the σs to octave intervals between 0.007 and 0.43 of the width of the stimulus. All 9 filters are separately convolved with the stimulus to produce 9 filters responses. Each filter response is then subject to response normalization. Response normalization here is implemented by multiplying each filter response by a constant, given by:

(2) $$\frac{1}{1+ka_{s}/\bar{a}}$$

where *a_s_* is the peak-to-mean amplitude of the filter response to the edge, *ā* is the mean amplitude across all filter scale responses and *k* is a constant that determines the strength of normalization. In all the simulations here *k* is fixed at 0.5 and was chosen to provide a good visual fit to the data from Figure 4 in Wallis and Georgeson (2012), shown here in Figure 7. Besides *k*, which is fixed throughout, there are no free parameters in the model. The effect of the response normalization stage is to reduce the response amplitudes of all filters, but more so for ones with relatively high amplitude, resulting in more equal filter responses across scale. The factor *ā* (mean amplitude) causes the normalization to be invariant with respect to stimulus contrast and results in Mach bands whose contrasts are invariant with stimulus contrast, as reported by Wallis and Georgeson (2012). It is important to note that the response normalization term used here is not the same as a saturating point-wise nonlinearity, such as the well-known Naka-Rushton equation (Naka and Rushton, 1966). The response normalization term here is a constant applied multiplicatively to each filter’s response, a constant that alters the amplitude of each response but does not “distort” its shape by introducing new Fourier components as does a point-wise saturating nonlinearity. In the final stage of the model the normalized responses of all 9 filters are summed to produce the predicted brightness profile. For illustration purposes the summed responses have been multiplied by another constant in order to scale them to match the amplitude of the stimulus luminance profile (0.4 in all cases), in order that the physical stimulus profile and its predicted appearance can be directly compared.

**Figure 4:**
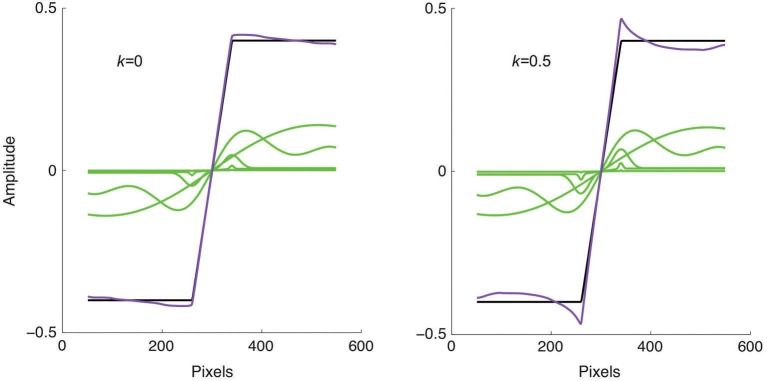
**Model applied to a trapezoidal edge, shown as the black line**. The green lines show the responses of four of the filter scales selected at 2 octave intervals, while the purple line shows the sum of all 9 filter responses. Left: without response normalization the summed response is close to veridical. Right: with response normalization Mach bands are produced.

**Figure 5:**
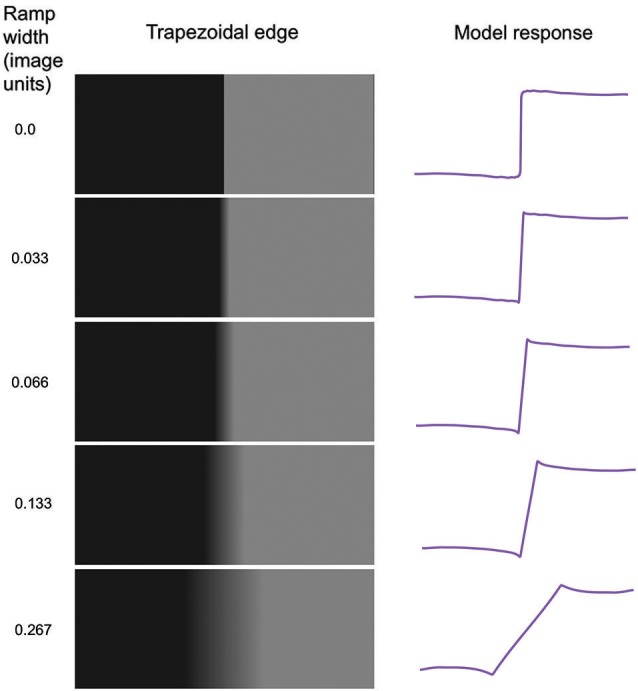
**Model applied to trapezoidal edges of various ramp widths**. Ramp width is given in units of image size.

**Figure 6:**
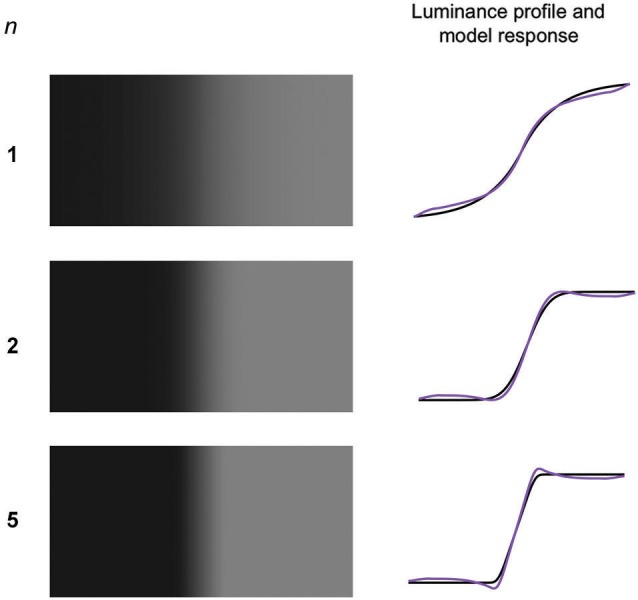
**Generalized Gaussian edges (GGEs) with three values of the exponent *n* and σ set to 0.067 of the width of the stimulus**. On the right are shown the stimulus luminance profiles (black) and model responses (purple).

**Figure 7:**
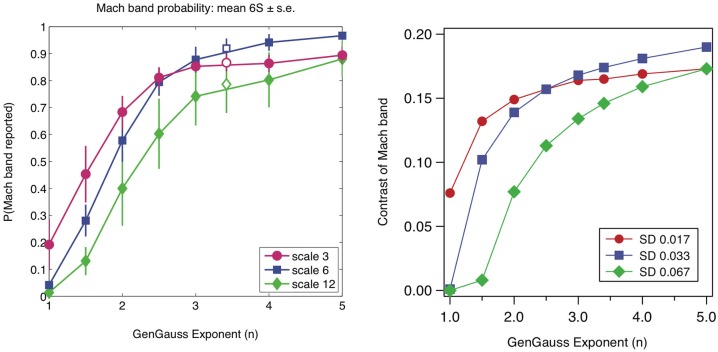
**Left: probability of seeing Mach bands in GGEs as a function of the exponent *n* and scale of edge (proportional to standard deviation σ): data from Figure 4 in Wallis and Georgeson (2012); reproduced with permission**. Right: predicted Mach band contrasts from the response normalization model for three values of standard deviation (SD) in image units, also at octave intervals.

Models of response normalization vary considerably in implementation. In some models the responses of each orientation and spatial-frequency of filter are divided at each point by the weighted sum of filter responses across all orientations (Carandini and Heeger, 2012) or scales (Robinson et al., 2007). In some cases the weighted sum itself is subjected to blurring across space using a Gaussian kernel (e.g., Robinson et al., 2007). Blakeslee and McCourt (1999) modeled response normalization by first pooling across scale the filter responses at each orientation, then dividing at each orientation the pooled response averaged across the whole stimulus. Conceptually, our model of response normalization is most similar to that of Dakin and Bex (2003), who convolved their stimuli with circularly-symmetric log Gabor filters and then set equal the amplitudes of the filter responses in order to produce stimuli with 1/*f* (where *f* is spatial-frequency) Fourier amplitude spectra. In our model the amplitudes are not set equal, just less unequal. The model here is constrained to being 1D, which rules out normalizing across orientation. Normalizing to response energy pooled across scale rather than to amplitude *within* a scale is an obvious alternative option. Suffice to say that the author’s attempts so far using cross-scale normalization, while successfully predicting the presence of Mach bands, have yet to produce results as good as the within-scale normalization employed here (in particular predicting the results shown in Figure 7). No doubt with the larger number of free parameters available with cross-scale normalization (e.g., the standard deviations of the Gaussian weighting functions across scale and across space, as well as the normalization constant) there is every reason to suppose that cross-scale normalization, especially in conjunction with 2D stimuli, may ultimately prove to be as good if not better a model than the one presented here.

## Results

The model output for a trapezoidal edge is shown in Figure 4, on the left with *k* = 0, i.e., with no response normalization, and on the right with *k* = 0.5. Why are Mach bands predicted only in the right hand figure? Although the foot and knee of the trapezoid elicit significant responses in the small-sized filters, their amplitudes are still lower than those from the large-sized filters that respond to the edge as a whole. The effect of response normalization is to relatively enhance these small-sized filters responses, resulting in the accentuated responses known as Mach bands.

### Effect of steepness of trapezoidal edge

Figure 5 shows trapezoidal edges with varying ramp widths, together with the predicted responses from the model. As can be seen the Mach bands become narrower as the ramp narrows, disappearing altogether in the limiting case of the step-edge. Although with the step-edge one observes a slight but broad brightening and darkening in the vicinity of the edge, this is not part of the continua that are Mach bands. Thus as has been previously reported (Fiorentini, 1972; Tolhurst, 1972; Ross et al., 1981; Ratliff, 1984) there are no Mach bands at a step edge. The model response profiles are shown on the right and accord with the percepts. The reason why no Mach bands are predicted by the model at a step-edge is simple. Because all scales of filters give the same amplitude of response to a step edge, no amount of normalization will impact upon their relative amplitudes, so the percept is predicted to be more-or-less veridical. Interestingly, the slight but broad brightening and darkening either side of the edge is captured by the model; it is caused by the fact that the filter set employed is not fully complete due to the absence of filters tuned to very low spatial frequencies.

### Generalized gaussian edges

In an elegant series of experiments, Wallis and Georgeson (2012) measured the frequency-of-seeing and positions of Mach bands in a class of edge termed “Generalized Gaussian edges”, or GGEs. The 1D formula for a GGE is:

(3) $$\begin{array}{l} L\left(x\right)=\int \exp\left(\frac{-{\left|x\right|}^{n}}{2\sigma^{n}}\right) \end{array}$$

where *L(x)* is the luminance profile and *x* is position. The parameter *σ* determines the width of the edge and *n* the sharpness of its transitions. When *n* is 2 the GGE is a conventional Gaussian edge, when *n* = 3.4 a sine-wave edge and when *n* is greater than about 8 the GGE approximates a trapezoid.

Example GGEs are shown in Figure 6 along with the model predictions. Wallis and Georgeson showed that observers very rarely reported Mach bands in GGEs with *n* < 2 and that the probability of seeing Mach bands increased monotonically with n for 1 > *n* <= 5. Figure 7 reproduces Wallis and Georgeson’s data, along with the predictions from the model, for which the normalization constant *k* of 0.5 that is used throughout this study was chosen to provide the best visual fit. For the model predictions the contrast of the Mach band is the dependent measure, defined as the difference in model response between the peak of the Mach band and the lowest part of the plateau either side, divided by the mean amplitude of the response. Although the two dependent measures, Mach band probability-of-seeing in Wallis and Georgeson, Mach band contrast here, are very different, the similarity between the data and model predictions is remarkable. The response-normalization model even predicts the switch in maximum frequency-of-seeing when going from scale 3 to scale 6 at *n* = 2.5.

### Phase-manipulated trapezoidal waveforms

**Figure 8:**
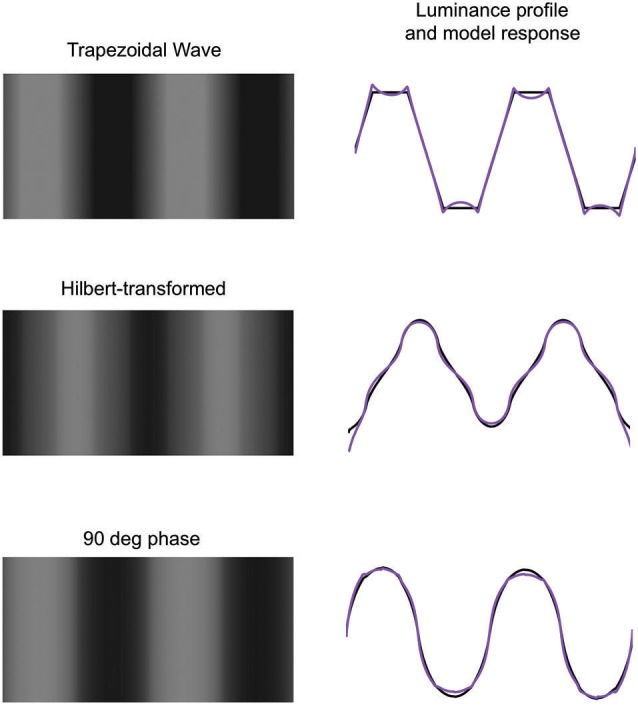
**Top left: trapezoidal waveform showing Mach bands**. Middle left: Hilbert transformed trapezoidal waveform. Bottom left: trapezoidal waveform with all Fourier phases set to 90 deg. On the right are shown the luminance profiles (black) and response-normalization model responses (purple).

In their study of Mach bands, Ross et al. (1989) manipulated the Fourier phase relationships of a trapezoidal waveform in order to determine the importance of local phase relationships for Mach bands. They demonstrated that whereas in a trapezoidal waveform Mach bands were visible, none were seen either when the waveform was subject to a Hilbert transform, which shifts all the Fourier phases by a quarter of a cycle, i.e., 90 deg, or when all the Fourier phases were set equal to 90 deg. Figure 8 confirms all three observations. Ross et al. concluded that Mach bands were observed only at points where the different Fourier components aligned in cosine phase, since the Hilbert transform re-aligned them instead in sine phase, and setting all the Fourier phases to 90 deg disrupted the cosine-phase alignment. Put another way, the two Fourier phase manipulations employed by Ross et al. effectively removed the sharply-discontinuous feet and knees of the waveform that normally elicit relatively strong responses in small-scale even-symmetric (i.e., cosine-alignment-sensitive) filters. Ross et al. argued that their findings were best explained in terms of the local energy Feature model (Morrone and Burr, 1988), in which relatively strong even-symmetric filter responses are interpreted as signaling the presence of bars. Figure 8 however shows that the response normalization model, while correctly predicting Mach bands in a trapezoidal wave, also correctly predicts no Mach bands in either type of phase-manipulated trapezoidal waveform.

### Effect of neighboring structure

**Figure 9:**
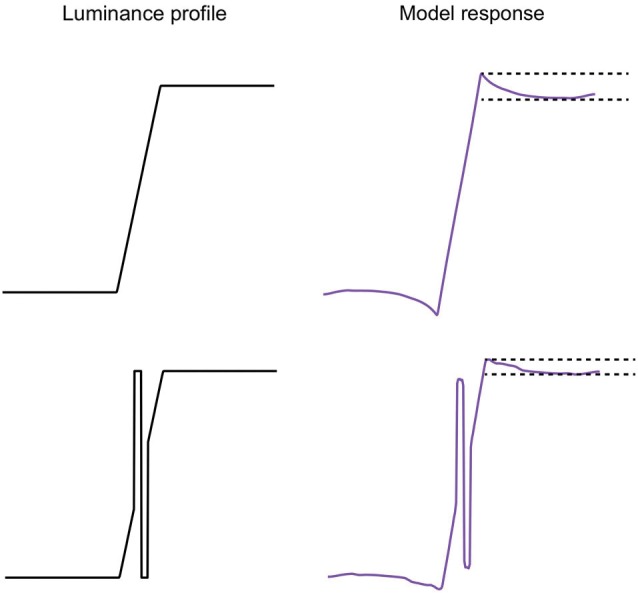
**Left: trapezoid luminance profile without (top) and with (bottom) a biphasic bar**. Right: model response. The separation of the pair of horizontal dashed lines in the model responses is a measure of the amplitude of the bright Mach band.

Ratliff et al. (1979, 1983) and Ratliff (1984) reported that Mach bands were attenuated by luminance contours positioned in the vicinity of the trapezoid. To explain this phenomenon, Ratliff (1984) invoked Tolhurst’s (1972) account of Mach bands in terms of interactions between edge and bar detectors. Ratliff (1984) suggested that the contour strongly stimulated edge detectors, and these tended to inhibit the nearly bar detectors that signaled the Mach bands at the foot and knee of the ramp. The effects of neighboring structure on Mach bands does not reproduce well on the printed page, so we have not attempted to show it here. Suffice to show that the response-normalization model predicts the phenomenon. Figure 9 shows how a biphasic bar positioned in the middle of a trapezoid (Ratliff, 1984) reduces the magnitude of the Mach bands in the model response. Why? In the normal trapezoid, the relatively small-scale filters that give significant responses to the foot and knee of the trapezoid are boosted by response normalization because they are nevertheless more weakly stimulated than the coarser-scale filters that respond to the trapezoid as a whole. The biphasic bar is a powerful stimulus to small-scale filters, enhancing their amplitudes and therefore making them less subject to the effects of response normalization. Hence weaker Mach bands.

## Discussion

We have shown that a bare-bones and simplified 1D model of response normalization provides a good account of the conditions in which Mach bands occur as well as when they do not occur. As such this modeling exercise represents a “proof-of-concept” alternative explanation of Mach bands to that of Feature models, as well as to the more recent models of Mach bands based on learnt image statistics (see below).

### Relationship to feature models

In the local energy (Morrone and Burr, 1988) and N2+1+ (Wallis and Georgeson, 2012) Feature models the edge-vs.-bar decision is based at each point of the image on the relative responses of even- and odd-symmetric filters, implying a divisive relationship between pairs of orthogonal-in-phase filter responses. This could be construed as a special type of response normalization, one in which one phase of filter response is “normalized” to that of another filter response of orthogonal phase. However to this author’s knowledge there is no physiological evidence for such phase-specific response normalization. Although the goals of Feature models and response normalization are arguably similar (both are presumably designed to achieve the efficient coding of visual information) the difference is that whereas in Feature models Mach bands are generated by a mechanism that is designed to categorize spatial luminance changes into edges and bars, in the response normalization model Mach bands are an emergent feature of a general-purpose nonlinearity designed for efficient coding.

Does feature coding play any role in Mach bands? If an observer is required to make explicit judgments about the bands, for example their position, width or apparent contrast, then presumably some sort of feature coding mechanism must be deployed. What is being argued here is that whatever this mechanism is, it is not the one that *generates* the Mach bands in the first place. The corollary to this argument is that it is sufficient to explain the *appearance* of Mach bands rather than have to explain how observers make explicit judgements about Mach band properties. This criterion of sufficiency is the same as that used to validate the models of illusory brightness phenomena that provided the inspiration for the present study (Blakeslee and McCourt, 1999; Dakin and Bex, 2003; Blakeslee et al., 2005; Robinson et al., 2007; Otazu et al., 2008). As with the model here, these models are content with predicting the brightness profiles of stimuli, obtained either from casual observation or from brightness matching procedures.

### Other explanations of Mach bands

Although the aim of this communication is not to review all explanations of Mach bands, it would be imprudent not to mention one other. In keeping with their “empirical” approach to visual illusions, Purves and Lotto (2003) argue that Mach bands are the result of learnt image statistics. They suggest that local luminance extrema similar in form to Mach bands are often found on curved surfaces that are subject to particular patterns of naturally-occurring illumination. As a result we have come to expect such luminance extrema, and so tend to perceive them as Mach bands even when they are not physically present. The challenge for the empirical approach will be to determine whether the variations observed in Mach band strength, size and position that have been reported here and elsewhere can be as easily explained by the physical luminance properties of images as by response normalization.

### Limitations of present study

This study has provided no new empirical data in support of the model it proposes. In contrast, Wallis and Georgeson’s (2012) N2+1+ Feature model of Mach bands was tested against a comprehensive series of measurements of the positions and visibilities of Mach bands across a range of stimuli and was found to give a very good account. The appeal of the response normalization model however lies in its simplicity and physiological plausibility. However the model needs to be tested against data for which the predictions are different from those of Feature models, so the challenge for the future will be to come up with those predictions.

## Conflict of interest statement

The author declares that the research was conducted in the absence of any commercial or financial relationships that could be construed as a potential conflict of interest.
